# Is a Depth Camera in Agreement with an Electromagnetic Tracking Device when Measuring Head Position?

**DOI:** 10.22599/bioj.227

**Published:** 2021-11-15

**Authors:** Vienna-Jaye Burchell, Gemma Arblaster, David Buckley, Jonathan Wheat

**Affiliations:** 1 Dorset County Hospital, GB; 2 University of Sheffield, GB; 3 Sheffield Hallam University, GB

**Keywords:** Polhemus, electromagnetic tracking system, Microsoft Kinect, depth camera, head position, abnormal head posture

## Abstract

**Introduction:**

Clinicians typically observe and describe abnormal head postures (AHPs) and may also measure them. Depth cameras have been suggested as a reliable measurement device for measuring head position using face-tracking technology. This study compared a depth camera (Microsoft Kinect) to a gold standard electromagnetic tracking system (Polhemus device) to measure head position.

**Method:**

Twenty healthy volunteers (mean age 21 years) had their head position simultaneously recorded using the depth camera (Kinect) and the electromagnetic tracking system (Polhemus). Participants were asked to make 30-degree head movements into chin up, chin down, head turn and head tilt positions. The head movement made and the stability of the head at each position were recorded and analysed.

**Results:**

Compared to the electromagnetic tracking system (Polhemus), the depth camera (Kinect) always measured a smaller head movement. Measurements with the two devices were not statistically significantly different for turn right (P = 0.3955, p > 0.05), turn left (P = 0.4749, p > 0.05), tilt right (P = 0.7086, p > 0.05) and tilt left (P = 0.4091, p > 0.05) head movements. However, the smaller depth camera measurement of chin up and chin down head movements were statistically significant, chin up (P = 0.0001, p < 0.01) and chin down (P = 0.0005, p < 0.001). At each eccentric position, the depth camera (Kinect) recordings were more variable than the electromagnetic tracking system (Polhemus).

**Conclusions:**

Compared to the electromagnetic tracking system (Polhemus), the depth camera (Kinect) was comparable for measuring head turns and tilts but was less accurate at measuring chin up and chin down head positions. Further research is needed before the depth cameras are considered for clinical recordings of head position.

## Introduction

Abnormal head postures (AHPs) may have an ocular or non-ocular aetiology; ocular causes include nystagmus and ocular motility problems (Nucci et al. 2005). AHPs due to uncorrected refractive error are typically eliminated upon correction of the refractive error (Nucci et al. 2005). AHPs consist of head tilt, face turn, chin up/down, or may be a combination of these. The aetiology cannot be predicted from an AHP, meaning that patients may be investigated by specialities including ophthalmology, neurology and orthopaedics (Nucci and Curiel 2009). Most clinicians use observation and written descriptions of head position, which can result in inter- and intra-observer variability. Errors of 2–18° have been reported when estimating AHPs by observation only (Kim et al. 2004), with an increased error for larger AHPs (Granet et al. 2001). Inaccurate AHP estimations make monitoring ocular conditions and measuring the effectiveness of treatments difficult, particularly if an AHP is the primary indication for management (Hald et al. 2011).

Different devices exist to measure and quantify head position and AHPs. The Cervical Range of Motion (CROM) device (approximate cost £500) can be used clinically. It is worn on the head and can be difficult to set up (Oh et al. 2014; Kim et al. 2012). It contains two inclinometers and one magnetometer on a shoulder mounted yoke to calculate three-dimensional head positions. It is not suitable for young children or uncooperative patients and training is required to use the device. However, measurement errors have been reported to be low for the CROM—1.6–2.8 degrees (Raya et al. 2018)— and it is reported to be accurate and comparable to an optoelectronic system (optotrak) (Tousignant et al. 2006). Other head position measuring devices use either fluid level devices, digital motion trackers or infrared optical trackers. Whilst these are reported to be user friendly, they require the subject to wear the device on a headband and they also rely on participant cooperation and operator experience (John et al. 2015; Kim et al. 2012; Hald et al. 2011).

Highly accurate electromagnetic tracking systems can be used to measure head position and are commercially available. These are more commonly used in research settings due to their high cost, challenging set up away from metallic structures, and the expertise needed for data analysis (Spitzley and Karduna 2019). During set up, anatomical points on each participant must be calibrated by, for example, using a stylus touched on the skin. Participants must also wear a head mounted sensor during recording (Alken Inc 2012). The Polhemus Liberty electromagnetic capture system (Polhemus, Colchester, VT, USA) is one example of an electromagnetic tracking system. It is considered to be a gold-standard measurement device used to obtain three-dimensional head and neck movement and position data, accurate to 0.15 degrees or 0.76 mm (Gudmundsson 2010; Nafis et al. 2006; Alken Inc 2012; Sjolander et al. 2008).

With improvements in low-cost imaging technology, remote noncontact cameras are increasingly being used to measure head position. Farah et al. (2018) used a mobile phone camera to measure abnormal head position and reported good accuracy, measuring a mean head position of 31.87 ± 0.81 degrees in 30° head postures. Thomas et al. (2016) used a webcam for the ‘Cambridge Face Tracker’, which showed good agreement with the CROM, with correlation coefficients of 0.96 or higher for 20–50° head movements. The Microsoft Kinect depth camera uses real time tracking of the face to provide instant measurements of head position up to 10 metres away (Oh et al. 2014; Toth et al. 2012). Oh et al. (2014) reported the Kinect had good reliability compared to the CROM, especially for head tilts and turns up to 30°. The largest difference between the Kinect and the CROM was 0.25–2.50° when measuring 30° chin up/down positions. Whilst the Kinect is low cost (around £200) and has shown comparable head position measurements to the CROM (Oh et al. 2014) it has yet to be compared to a gold standard head position measurement device. The aim of this study was to compare a depth camera (Kinect) to a gold-standard electromagnetic tracking system (Polhemus device) to measure head position.

## Methods

### Participants

Ethical approval was granted from the University of Sheffield. Undergraduate students were recruited to the study. The inclusion criteria were monocular visual acuity of 0.100 logMAR or better (crowded logMAR at 3 metres), no manifest deviation, near point of convergence 10 cm or better, no ocular motility defect, no AHP, and no history of ocular pathology. Participants with refractive errors were required to wear contact lenses to be included, due to the potential for metal in glasses to cause inaccurate measurements with the electromagnetic tracking system (Polhemus).

### Experimental setup

**Figure 1:**
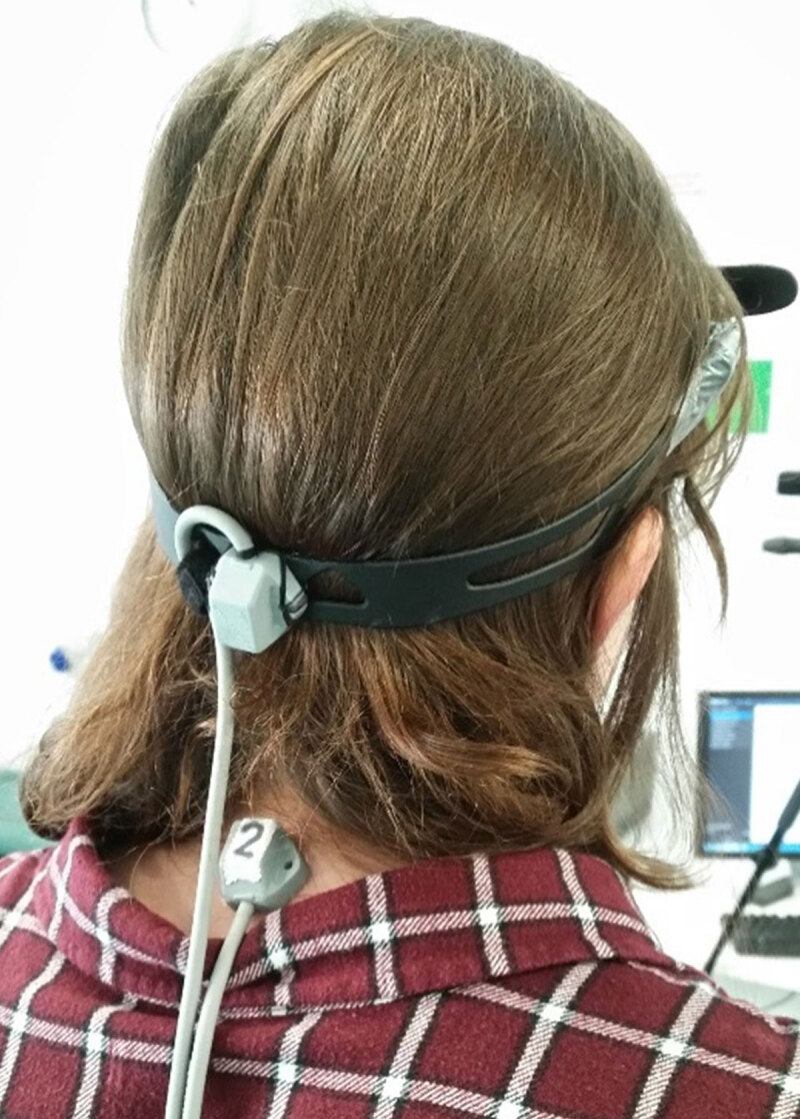
A participant wearing the adjustable headband with the head sensor attached, as well as the torso sensor attached to the participant’s base of the neck with double-sided tape.

**Figure 2:**
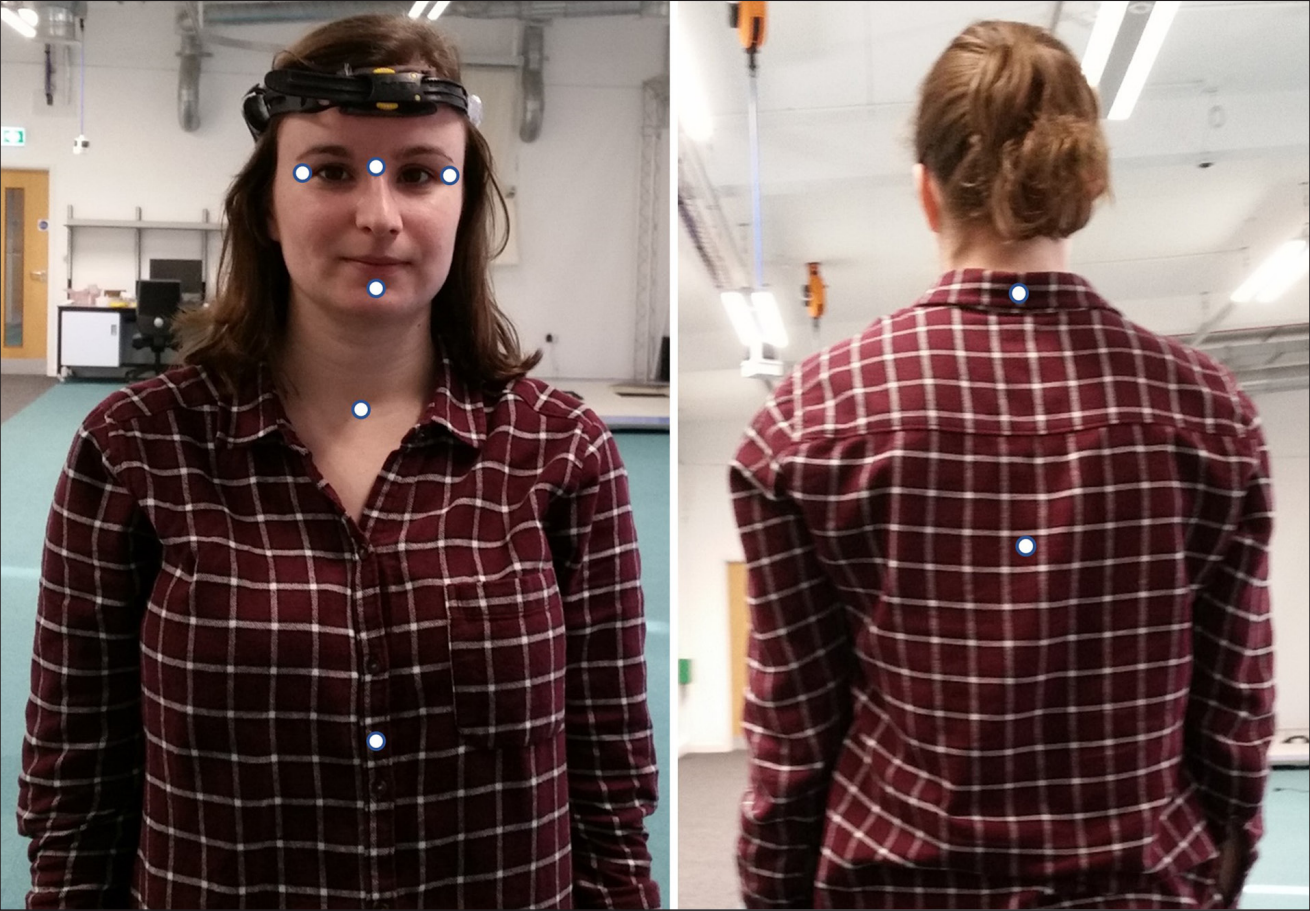
The anatomical points shown as white dots that were calibrated with the stylus as shown in Figure 2. The anatomical points that were calibrated were: central glabella, orbital margin lateral to right and left lateral canthi, 1 cm below bottom of bottom lip, sternal notch and xiphoid process, 7^th^ cervical vertebrae and 8^th^ thoracic vertebrae.

Two Polhemus system sensors were firmly attached to the back of the head (using a plastic strap) and to the base of the neck (using medical tape) (***Figure 1***). A digital stylus was used to locate the anatomical landmarks within the relevant sensor’s reference frame (central glabella, orbital margin lateral to right and left lateral canthi, 1 cm below bottom of lower lip, sternal notch, xiphoid process, 7^th^ cervical vertebrae and 9^th^ thoracic vertebrae) (***Figure 2***). These points corresponded to those detected with the Kinect and were compared during analysis.

All recordings took place in a bright, white and well-lit room. Participants were instructed to stand 1.50 metres directly in front of the depth camera (Kinect), which was mounted on a tripod 1.50 metres from the floor. Fixation targets were placed on a wall in a central position, 30 degrees horizontally (to the right and the left), 30 degrees vertically (above and below the central target) and tilted 30 degrees (in a central position). The fixation targets were rectangles with a central cross of a large enough size that the devices did not obscure the targets.

Participants were instructed to keep their body still and move their head slowly and steadily to fixate on each target in turn for 10 seconds. After the head movement to each eccentric target, participants returned to fixate on the central target for 10 seconds. Head movements were performed in the same order (chin up, chin down, turn right, turn left, tilt right, then tilt left). Participants were observed during the task to ensure they were making the required head movements and were not keeping their head still and moving their eyes to fixate on each target. If eye movements were observed, the data for that movement was deleted, the participant was reminded to move their head instead of their eyes, and the movement was repeated.

The depth camera (Kinect) and the electromagnetic tracking system (Polhemus) simultaneously measured 3-dimensional head and torso position and orientation, in their respective global coordinate systems.

### Analysis

Equivalent data points on the face were used for both the depth camera (Kinect) and the electromagnetic tracking system (Polhemus). For the electromagnetic tracking system (Polhemus), a local head coordinate system was defined using three anatomical points (orbital margin lateral to right and left lateral canthi, and 1 cm below bottom of lower lip).

Briefly, the origin of the coordinate system was at the midpoint between the orbital margins between the right and left lateral canthi, the x-axis was mediolateral pointing to the participants’ right (coincident with the vector passing from the origin to the orbital margin lateral to right lateral canthi), the y-axis was perpendicular to the plane formed by the origin of the coordinate system, orbital margin lateral to right lateral canthi and the point 1 cm below bottom of lower lip, and the z-axis was mutually orthogonal to the x- and y-axes. For the depth camera (Kinect) the points returned by the Kinect that were closest to the Polhemus points were used. Euler angles were calculated to define the orientation of the head (rigid body) relative to the world using a fixed coordinate system, from the orientation matrix defined from the head coordinate system unit vector, using a xyz sequence. Euler angles x, y and z represent chin up/down, head turn and head tilt, respectively.

Raw data was processed using MATLAB. The processed data was tabulated and displayed graphically. The measurement of the range of each head movement and the stability of the head position whilst fixating on a target for 10 seconds were extracted for both depth camera (Kinect) and the electromagnetic tracking system (Polhemus). For the stability of head position data, whilst each participant was asked to maintain the eccentric position for 10 seconds, only the middle 8 seconds of data was included in the analysis. The Kinect generated approximately 30 frames per second, compared to the Polhemus with a higher sample rate of approximately 240 frames per second. During the middle 8 seconds of data collection for each head movement, around 240 data points were analysed from the Kinect, compared to 1920 data points from the Polhemus. To analyse the range of head movement, the Wilcoxon matched-pairs signed rank test was used to compare the measurements from the depth camera (Kinect) and the electromagnetic tracking system (Polhemus) for each head position. To analyse the stability of the head movement, the paired t-test was used to compare the standard deviations of the recordings from the depth camera (Kinect) and the electromagnetic tracking system (Polhemus) for the chin up, chin down, turn left and tilt left head positions.

## Results

Twenty-one participants were recruited to the study. One participant was excluded for failing to meet the visual acuity criteria. Head position data was recorded simultaneously for twenty participants using both the depth camera (Kinect) and the electromagnetic tracking system (Polhemus). No technical problems occurred, and no data was excluded from the electromagnetic tracking system (Polhemus). The depth camera (Kinect) experienced some problems with continuous real time face tracking. If face tracking was lost during a recording, depth camera (Kinect) data was excluded for that particular head movement, as well as the corresponding electromagnetic tracking system (Polhemus) data.

### Range of head movement

**Figure 3:**
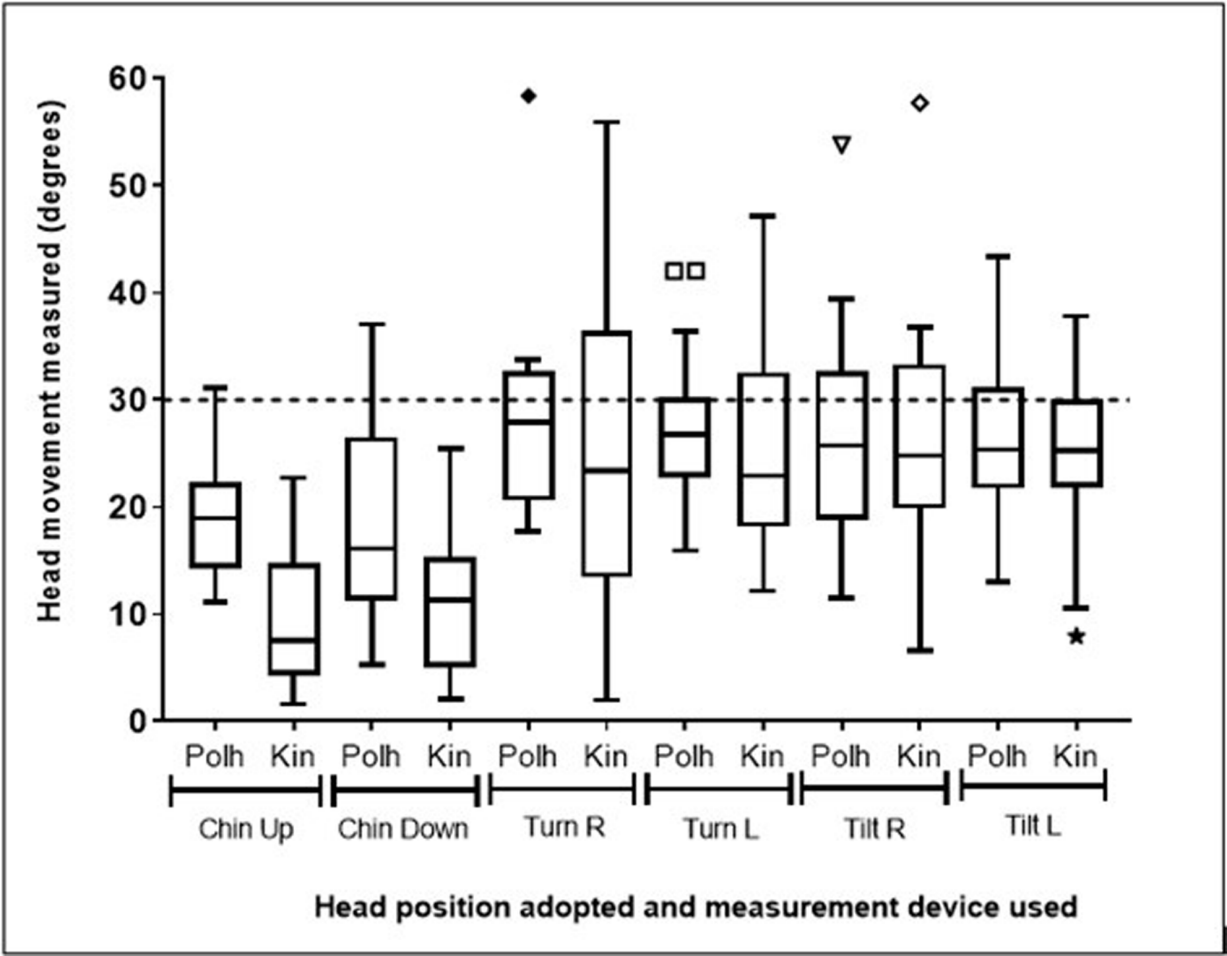
A box and whisker plot (Tukey method) showing the median and IQR measurements (degrees) of head movement to each eccentric position. Dashed line represents the 30-degree movement participants were asked to make to fixate on each target. (Polh = Polhemus, Kin = Kinect, R = right, L = left).

The median and interquartile range of the head movements made by all participants are shown in ***Figure 3***.

The depth camera (Kinect) measured less head movement than the electromagnetic tracking system (Polhemus) for all head movements made except tilt left which was similar. The difference between the depth camera (Kinect) and the electromagnetic tracking system (Polhemus) measurements was statistically significant for the chin up (p = 0.0001) and chin down (p = 0.0005) movements. The differences between the depth camera (Kinect) and the electromagnetic tracking system (Polhemus) measurements were not statistically significant for the turn right (p = 0.3955), turn left (p = 0.4729), tilt right (p = 0.7086) and tilt left (p = 0.4091) movements.

### Stability of head movement

**Figure 4:**
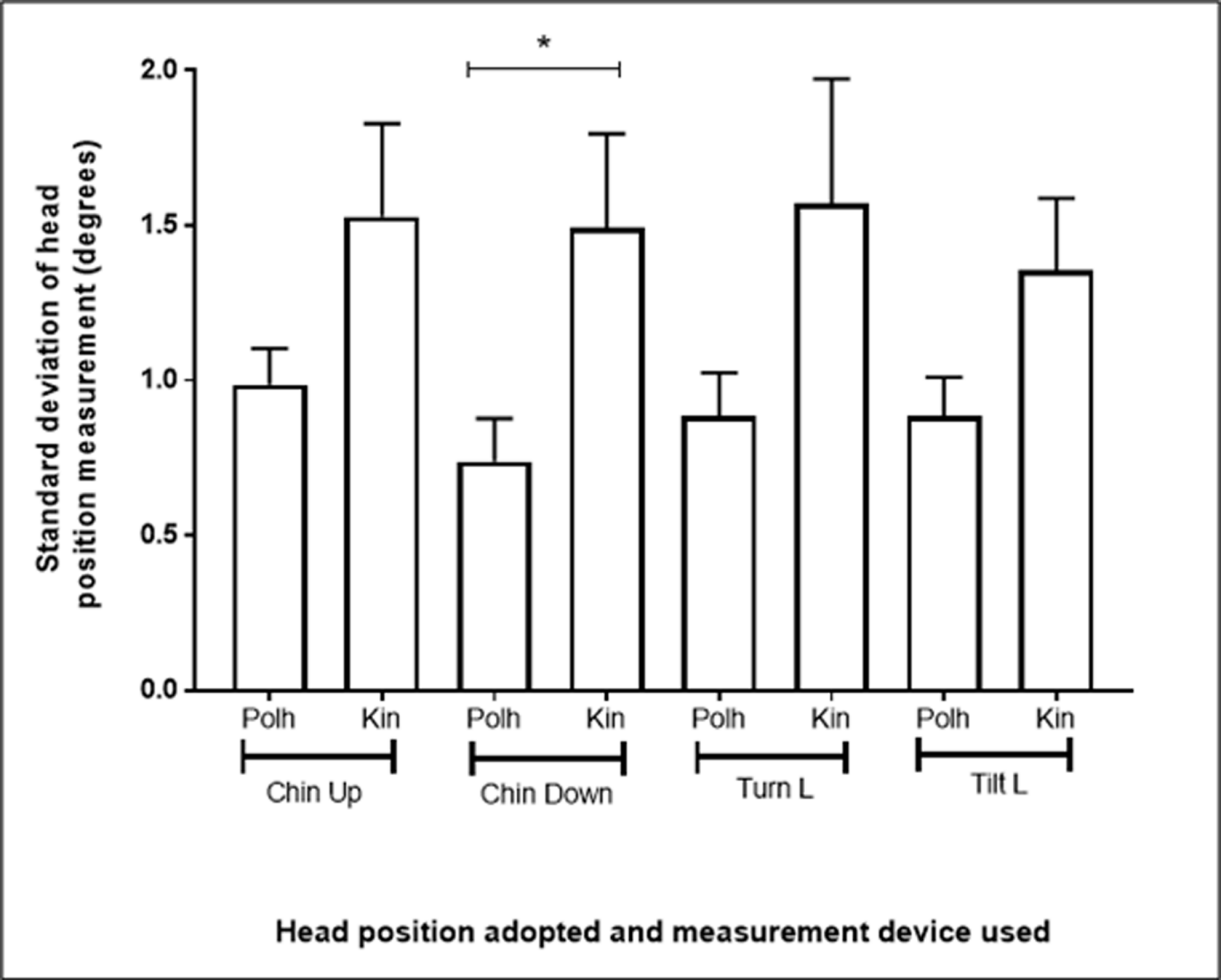
A bar chart showing the standard deviation and standard error of the measurements from the Polhemus and the Kinect when each eccentric head position was maintained for 8 seconds. (* denotes statistically significant difference). Polh = Polhemus, Kin = Kinect, R = Right, L = Left.

The mean standard deviation of the head position measurements was calculated and is shown in ***Figure 4***.

Due to the difference between the chin up and chin down measurements, the stability data for both of these positions is included. Data for turn left and tilt left head movements are included, as there was little difference between turn right and turn left, and tilt right and tilt left measurements. The standard deviations of the measurements recorded with the depth camera (Kinect) and the electromagnetic tracking system (Polhemus) were not statistically significantly different for the chin up (p = 0.0882), turn left (p = 0.0884), or tilt left (p = 0.0990) head positions. The standard deviation of the depth camera (Kinect) measurements (1.49 degrees) during the chin down head position was larger than the standard deviation of the electromagnetic tracking system (Polhemus) measurements (0.74 degrees) and the difference between these standard deviations was statistically significant (p = 0.026).

## Discussion

The depth camera (Kinect) consistently measured a smaller head movement than the electromagnetic tracking system (Polhemus). The depth camera (Kinect) measurement was comparable to the electromagnetic tracking system (Polhemus) for head turn right and left, and head tilt right and left; however, there was a greater difference between the measurements from the two devices for chin up and chin down head movements.

### Range of Movement

The depth camera (Kinect) had good agreement with the electromagnetic tracking system (Polhemus) for measuring head turns and tilts with an average underestimation of 4.50° for turn right, 3.80° for turn left, 1.00° for tilt right and 0.04° for tilt left. These underestimations were not statistically significant and are unlikely to be clinically significant. This was similar to the results of Oh et al. (2014), who found the Kinect to correlate closely with the CROM, with a mean angular difference of 0–0.88° for head turns and 0.04–1.75° for head tilts between the two devices. In this study, the depth camera (Kinect) had poorer agreement with the electromagnetic tracking system (Polhemus) for sagittal plane head movements, with an average underestimation of 11.37° for chin up, 4.82° for chin down. This is likely to be due to the facial features being less visible to the depth camera (Kinect) in a 30° chin up and chin down position. Oh et al. (2014) also found the largest difference between the Kinect and the CROM for chin up and chin down head positions, however they reported much smaller differences (0.25–2.50°). The smaller differences found by Oh et al. (2014) may be due to their experimental setup being adjusted to each participant’s height. Oh et al. (2014) measured head movements every 10 degrees, up to 30 degrees, for head turns and chin up/down movements, and up to 40 degrees for head tilts. Our single measurement at 30 degrees of head movement precluded a calculation of the correlation between the two devices, yet it did allow a comparison of the two measurements at a large abnormal head position. Further measurements at different angles, such as 10, 20, 30 and 40 degrees, would allow further comparison, but were not performed due to time constraints. Oh et al. (2014) did not find an incremental increase in errors with increasing AHP.

Oh et al. (2014) reported difficulties with Kinect facial tracking, although particularly for more complex head positions (combination of chin up or down, turn and tilts). They eliminated data with less than 50% facial recognition, although they do not state how much data had to be discarded from their analysis. We did not specifically measure facial recognition rate or exclude data for this reason, however if the depth camera (Kinect) was unable to recognise sufficient facial features it did not record data. Nevertheless, it is possible that excluding some data with a lower recognition rate may have improved the accuracy of our measurements of chin up and chin down head positions.

Oh et al. (2014) positioned each participant’s head into an eccentric position before recording head position with the Kinect and the CROM separately. Whilst this allowed them to measure larger head positions, it is possible that there was a difference in the placement of the head position between measurements with the two devices using this technique. During our study, participants were asked to move their head and look directly at eccentrically positioned fixation targets whilst the recordings from the two devices were taken simultaneously. This is the likely reason for the smaller head movements made by participants, however the measurements taken from the two devices are of the same head position.

### Stability of movement

During each sustained eccentric head position, a small amount of variability in head position was recorded with the depth camera (Kinect) (1.36–1.53°) and the electromagnetic tracking device (Polhemus) (0.74–0.99°). Only the 0.75° difference in the stability of the chin down head movement between the depth camera (Kinect) and the electromagnetic tracking device (Polhemus) was found to be statistically significant (p = 0.026), however this is unlikely to be clinically significant. Stability of head movement at eccentric positions has not previously been reported for the depth camera (Kinect). Oh et al. (2014) instead measured test-retest variability for the Kinect and reported good limits of agreement (–5.30–+4.98°) for 30° head position measurements.

It is acknowledged that this study included volunteers with no AHPs, however evidence in ‘visually normal’ participants is important to establish the reliability of new technology before it is considered for use in clinical populations. It may be that subjects with AHPs may have reduced variability of measurements as they have a visual need to maintain their head posture. Future studies of head position should consider moving the head to an eccentric position, as we found participants made smaller head movements than the required 30-degree movements. Further work to refine the optimum setup of depth cameras, such as the Kinect, would be beneficial to ensure maximally accurate measurements are recorded, particularly for chin up and chin down measurements, where less of the facial features can be seen.

Remote digital depth cameras, such as the Kinect, have potential to be used for measuring head positions, due to the ease of set up and relatively low cost (Thomas et al. 2016). Whilst the depth camera we used (Kinect) underestimated all head movements made by a range of 0.04° to 11.37° compared to the electromagnetic tracking device (Polhemus), the differences for face turns and tilts were small and unlikely to be clinically significant. The differences between the two devices were larger for chin up and down measurements, yet it is not known whether these differences would be clinically significant. Further work to measure head position in clinical populations would help establish the size and range of AHPs that are adopted. In addition, comparisons of current clinical estimations of head position and the Kinect would be beneficial, as depth cameras, such as the Kinect, may be more accurate than observation alone, which has been reported to be inaccurate by up to 30° (Kim et al. 2004).

## Conclusion

There is potential for depth cameras, such as the Kinect, to be used to measure head position as they are relatively cheap and non-contact. Whilst depth cameras are easy to use, the processing and analysis of the data generated currently requires expertise. The depth camera (Kinect) was comparable to the electromagnetic tracking system (Polhemus) when measuring head tilts and turns. Differences between the depth camera (Kinect) and electromagnetic tracking system (Polhemus) measurements of chin up and down movements were statistically significant but may not be considered clinically significant. Both the depth camera (Kinect) and electromagnetic tracking system (Polhemus) measure a small amount of variability when an eccentric head position is maintained, which may not be clinically significant.
